# Health-Based Cyanotoxin Guideline Values Allow for Cyanotoxin-Based Monitoring and Efficient Public Health Response to Cyanobacterial Blooms

**DOI:** 10.3390/toxins7020457

**Published:** 2015-02-05

**Authors:** David Farrer, Marina Counter, Rebecca Hillwig, Curtis Cude

**Affiliations:** Public Health Division, Oregon Health Authority, 800 NE Oregon Street, Suite 640, Portland, OR 97232, USA; E-Mails: marina.counter@state.or.us (M.C.); rebecca.hillwig@state.or.us (R.H.); curtis.g.cude@state.or.us (C.C.)

**Keywords:** cyanotoxins, anatoxin-a, cylindrospermopsin, microcystin, saxitoxins, drinking water, recreational water

## Abstract

Human health risks from cyanobacterial blooms are primarily related to cyanotoxins that some cyanobacteria produce. Not all species of cyanobacteria can produce toxins. Those that do often do not produce toxins at levels harmful to human health. Monitoring programs that use identification of cyanobacteria genus and species and enumeration of cyanobacterial cells as a surrogate for cyanotoxin presence can overestimate risk and lead to unnecessary health advisories. In the absence of federal criteria for cyanotoxins in recreational water, the Oregon Health Authority (OHA) developed guideline values for the four most common cyanotoxins in Oregon’s fresh waters (anatoxin-a, cylindrospermopsin, microcystins, and saxitoxins). OHA developed three guideline values for each of the cyanotoxins found in Oregon. Each of the guideline values is for a specific use of cyanobacteria-affected water: drinking water, human recreational exposure and dog recreational exposure. Having cyanotoxin guidelines allows OHA to promote toxin-based monitoring (TBM) programs, which reduce the number of health advisories and focus advisories on times and places where actual, rather than potential, risks to health exist. TBM allows OHA to more efficiently protect public health while reducing burdens on local economies that depend on water recreation-related tourism.

## 1. Introduction

In August of 2009, a series of dog deaths occurred along the South Umpqua River in Douglas County, Oregon. One of those deaths was confirmed to be the result of exposure to a toxin produced by certain genera of photosynthetic cyanobacteria, also called blue-green algae. The deceased dog’s stomach contents contained 10 µg/L anatoxin-a. In August of 2010, another dog death was confirmed to be caused by exposure to anatoxin-a. This dog, a healthy six month old black Labrador retriever, was vomiting, staggering, and convulsing within 10 min of drinking and playing in water from an isolated pool along the banks of the same stretch of the South Umpqua River and was dead within an hour. The treating veterinarian reported that her hands were “burning” after handling the dog’s body.

A subset of cyanobacterial genera includes member species that are capable of producing toxins, known as cyanotoxins. Cyanotoxin production is not universal or constant even among those species and strains that carry the necessary genes. The conditions that induce cyanotoxin production in capable species have not been elucidated [1]. Under certain environmental conditions that have not been conclusively defined, cyanobacteria can proliferate to form blooms consisting of significant biomass and covering large areas in fresh or marine water [1]. When blooms are dominated by potentially toxigenic genera of cyanobacteria, they are referred to as harmful algae blooms (HABs). 

By 2009, HABs composed of genera capable of producing at least four cyanotoxins (anatoxin-a, cylindrospermopsin, microcystins, and saxitoxins) had been identified in fresh bodies of water in Oregon. The anatoxin and saxitoxin families of cyanotoxins are primarily neurotoxic and are structurally described as alkaloids [1]. Anatoxin-a has a molecular weight of 165 daltons and exerts neurotoxicity by binding the neuronal pre-synaptic acetylcholine receptor with higher affinity than either nicotine or acetylcholine [1]. This binding leads to potent and prolonged nerve depolarization, which prevents further impulse transmission and at sufficient doses produces paralysis, asphyxiation, and death [1]. Saxitoxins make up a large family of alkaloids that are all larger than anatoxin-a and are the causative agents in human paralytic shellfish poisoning [1]. Saxitoxins block sodium ion channels in the axonal membranes of nerve cells [1]. This blockage prevents nerve impulse generation and propagation and at sufficient doses can cause paralysis, asphyxiation, and death [1]. Microcystins are a family of structurally related cyclic peptides. All microcystins consist of seven amino acids [1], but there are at least 80 structural variants, or congeners [2]. Microcystins induce cytotoxicity by inhibiting phosphatase activity [1]. Microcystins are primarily hepatotoxic because the cell membrane transporters required for cells to take up microcystins are most abundant in the liver [1]. Cylindrospermopsin is a cyclic guanidine alkaloid with a molecular weight of 415 daltons [1]. In its pure form, cylindrospermopsin primarily targets the liver, but crude extracts from cyanobacteria that produce cylindrospermopsin also affect the kidney, spleen, thymus, and heart [1]. The mechanism of toxicity appears to be inhibition of protein synthesis [1].

At the time of this manuscript’s submission, no U.S. federal standards or criteria have been published for any cyanotoxins. The World Health Organization (WHO) has established a suggested drinking water guideline value of 1 µg/L and a recreational exposure guideline value of 10 µg/L for a single variant of microcystin called microcystin-LR [1]. Health Canada has also published a drinking water standard of 1.5 µg/L for microcystin-LR [3]. 

The Oregon Health Authority (OHA) Public Health Division needed guidelines for meaningful, health-based evaluation of measured cyanotoxin data for all cyanotoxins that occur in Oregon. Drawing from peer-reviewed literature, standard risk assessment methodologies, and efforts of other states and nations, OHA developed provisional guideline values for anatoxin-a, cylindrospermopsin, microcystins and saxitoxins for drinking and recreational water use for humans and recreational water use for dogs. These guidelines allowed for implementation of toxin-based monitoring (TBM) by partner agencies and a more refined public health response. 

## 2. Methods

### 2.1. Development of Guideline Values

#### 2.1.1. Guideline Derivation and Exposure Assumptions for Humans

OHA independently reviewed toxicity studies in the literature and government reviews of those studies to identify no observable adverse effect levels (NOAELs), lowest observable adverse effect levels (LOAELs) or benchmark doses (BMDs) that could be used as a point of departure (POD) for calculation of a tolerable daily intake (TDI). TDIs were calculated from PODs by applying uncertainty factors (UF) (see Equation (1)).

(1) $$\text{TDI}=\frac{\text{POD}}{\text{UF}}$$

UFs provide a margin of safety in the presence of scientific uncertainties about the applicability of the underlying toxicity studies to the general human population. The most common types of uncertainty that require UFs are interspecies variability, individual variability, and limitations in the toxicological database. Interspecies UFs account for the fact that animals used in toxicity studies may differ from humans in the ways they absorb, distribute, metabolize, or excrete toxins. They may also differ from humans in their ability to repair damage caused by toxins. Individual variability UFs account for the fact that humans could have considerable individual variability in their sensitivity to cyanotoxins. For example, a child may be more sensitive than an adult, or people with particular genetic traits may be more sensitive than the general population. UFs for database limitation indicate a paucity of relevant toxicity studies or toxicological endpoints evaluated in those studies, reflecting the possibility that additional studies or endpoints could identify a lower dose with adverse effect than current studies have characterized.

Once TDIs were established, OHA applied exposure factors to calculate guideline values applicable for drinking water and recreational use in humans and for recreational use in dogs. The exposure factors OHA considered were body weight (BW), oral intake rate (IR) and relative source contribution (RSC), as shown in Equation (2). Government agencies commonly apply RSC when developing drinking water guideline values to account for other sources of a given contaminant, other than drinking water, in an individual’s overall exposure.

(2) $$\text{Guideline Value }=\frac{\text{TDI}\times \text{BW}\times \text{RSC}}{\text{IR}}$$

Table 1 describes the exposure factors that OHA used to develop drinking water and recreational guideline values for humans. As described above, TDIs are specific to individual cyanotoxins. OHA elected to use the WHO’s default BW of 60 kg for drinking water [1] as opposed to the EPA’s 70 kg [4] to be more protective of public health. For recreational water use, OHA chose a BW of 20 kg to represent children 4 to 6 years old based on data from the EPA [4]. OHA considered that 4 to 6 year olds would be the youngest age group likely to be swimming up to 2 h per day. OHA selected an RSC of 1, assuming 100% of an individual’s cyanotoxin intake would be through water swallowed either as drinking water or incidentally through recreational water use. For drinking water, OHA used EPA’s and WHO’s default assumption of 2 liters per day for an adult IR [1,4]. For recreational use, OHA used a default IR of 0.05 liters per hour of swimming from the U.S. Agency for Toxic Substances and Disease Registry’s (ATSDR) public health assessment guidance manual [5]. OHA further assumed that a child might swim for up to 2 hours per day in affected water, thus, 0.05 liters per hour equals 0.1 liters per day. 

**Table 1:** Exposure factors to calculate human drinking and recreational water guidelines.

Exposure factor name	Drinking water	Recreational water	Units
Tolerable daily intake (TDI)	Cyanotoxin dependent	Cyanotoxin dependent	Micrograms cyanotoxin per kilogram body weight per day (µg/kg-day)
Body weight (BW)	60	20	Kilograms (kg)
Relative source contribution (RSC)	1	1	Unitless
Intake rate	2	0.1	Liters per day (L/day)

#### 2.1.2. Guideline Derivation and Exposure Assumptions for Dogs

OHA calculated dog-specific guideline values using the same TDIs (with the exception of saxitoxins) as for humans but dog-specific exposure factors developed by California’s Office of Environmental Health Hazard Assessment (CALOEHHA) [6]. Because BW is so variable among different breeds of dogs, there is no single assumption that could be used with any certainty. CALOEHHA developed a BW-normalized water intake rate of 0.255 L of water per kilogram body weight per day (L/kg-day) for exercising dogs [6]. Therefore, OHA used a BW-normalized water IR (BWIR) of 0.255 L/kg-day in Equation 3 to calculate dog-specific guideline values.

(3) $$\text{Guideline Value}=\frac{\text{TDI}}{\text{BWIR}}$$

### 2.2. Derivation of Tolerable Daily Intakes

#### 2.2.1. Anatoxin-a

OHA reviewed available literature on the toxicology of anatoxin-a [1,7,8,9,10,11,12,13,14,15,16,17] as well as accepted and proposed threshold values used in other governmental jurisdictions [18,19,20,21,22]. OHA selected a study conducted by Fawell *et al.* [13,14] as the critical study for derivation of a TDI. In this study, groups of 10 male and 10 female mice were orally treated with anatoxin-a every day for 28 days at 4 doses (0, 100, 500 and 2500 µg/kg-day). The mice were observed for survival, clinical signs of toxicity or illness, and changes in body weight and food consumption throughout the course of the study. Eye function was evaluated at the beginning and end of the study, and hematology and serum biochemistry were evaluated in the last week of the study. Upon necropsy, Fawell *et al.* [13,14] examined all mice for gross lesions and examined all tissues from all mice histologically. Mice that died prior to the end of the study were also necropsied for gross lesions, and histological evaluations were performed. 

Three animals died during the study. One death, not related to treatment, resulted from animals fighting in their cages. There were two additional deaths, one at 500 µg/kg-day and one at 2500 µg/kg-day. The authors of the study and EPA reviewers indicated that the death at 500 µg/kg-day was not likely associated with treatment [13,14,20]. However, absent any other explanation for the death, OHA decided to assume that the death at 500 µg/kg-day was treatment related and used this as the LOAEL. None of the surviving animals had any observable adverse health effects by the evaluation methods employed, and there were no deaths at the 100 µg/kg-day dose. OHA therefore selected 100 µg/kg-day as the NOAEL and POD for TDI calculation. 

OHA applied a total UF of 1000 resulting in a TDI of 0.1 µg/kg-day using the formula shown in Equation 1. This UF is a composite of three types of uncertainty associated with this POD. First, an uncertainty factor of 10 was applied to account for interspecies differences in toxicity between mice and humans. Another UF of 10 was applied to account for individual variability within the human population. Finally, OHA applied an additional UF of 10 due to limitations in the database.

#### 2.2.2. Cylindrospermopsin

OHA selected the EPA’s proposed subchronic oral reference dose (RfD) of 0.03 µg/kg-day [23] as the TDI for cylindrospermopsin. EPA’s proposed RfD is based on an 11-week study in mice by Humpage, *et al*. [24] in which groups of male Swiss albino mice were dosed with 0, 30, 60, 120, or 240 µg/kg-day (10 mice per dose group) of purified cylindrospermopsin by daily gavage. Authors monitored food and water consumption and body weights throughout the study. Nine weeks into the study, authors conducted clinical exams with a focus on physiological and behavioral signs of toxicity. An extensive panel of parameters was measured in serum and urine near the end of the study along with hematological endpoints. Upon necropsy, organs were weighed and all tissues were examined histologically. No deaths were reported in the study. The most sensitive endpoint observed was kidney weight, which increased in a dose-dependent manner starting at 60 µg/kg-day [24]. The EPA selected 60 µg/kg-day from this study as the LOAEL and 30 µg/kg-day as the NOAEL [23]. 

EPA used a linear fit benchmark dose model to calculate a benchmark dose level of 33.1 µg/kg-day using the kidney weight data from all but the highest dose group in the critical study [23]. EPA used 33.1 µg/kg-day as their POD [23]. EPA then applied a total UF of 1000 resulting in an RfD of 0.03 µg/kg-day (with rounding) while noting that application of the same uncertainty factor to the NOAEL of 30 µg/kg-day would have yielded the same RfD [23]. The total UF of 1000 was a composite of an UF of 10 for interspecies variability, 10 for individual variability, and 10 for database limitations [23].

#### 2.2.3. Microcystins

Microcystins are cyclic heptapeptides with approximately 80 known structural variants [2]. These variations have significant influence on the toxicity and physio-chemical properties of the toxin. Microcystin-LR is the only variant for which there is sufficient toxicity data to develop a TDI.

OHA selected a study by Heinze *et al*. [25] as the critical toxicity study for derivation of a TDI for microcystin-LR. In this study, researchers treated rats with purified microcystin-LR in drinking water for 28 days and then measured body weights and weights of the liver, kidney, adrenal glands, thymus and spleen. Heinze *et al.* [25] also assessed hematology, measured an extensive list of parameters in serum, and examined all tissues microscopically for histopathology [25]. The Heinze study identified a LOAEL of 50 µg/kg-day for intrahepatic hemorrhage.

OHA used the LOAEL identified in the Heinze study [25] described above (50 µg/kg-day) as the POD to calculate a TDI of 0.05 µg/kg-day by applying a total UF of 1000 (10 for LOAEL to NOAEL adjustment, 10 for interspecies variability and 10 for individual variability) as described by Equation (1).

#### 2.2.4. Saxitoxins

The saxitoxin family of toxins includes saxitoxin (STX), neosaxitoxin (neoSTX), gonyautoxins, (GTX), C-toxins (C), 11-hydroxy-STX and decarbamoylsaxitoxins (dcSTXs) [17]. Because individual STXs vary in their toxicity, the European Food Safety Authority (EFSA) has developed toxic equivalency factors (TEFs), based on toxicity, in mice, so individual toxin concentrations can be considered relative to the toxicity of STX [26]. The proposed TEFs are: STX = 1, neoSTX = 1, GTX1 = 1, GTX2 = 0.4, GTX3 = 0.6, GTX4 = 0.7, GTX5 = 0.1, GTX6 = 0.1, C2 = 0.1, C4 = 0.1, dc-STX = 1, dc-neoSTX = 0.4, dc-GTX2 = 0.2, GTX3 = 0.4 and 11-hydroxy-STX = 0.3 [26]. OHA adopted these TEFs to develop a TDI for STX-equivalents (STX-eq). 

OHA selected a study by the EFSA [26] as the critical study for derivation of a TDI. EFSA established an acute RfD for STX-eq of 0.5 µg STX-eq/kg-day [26]. This acute RfD is based on available intoxication reports in humans across the European population. This acute RfD represents an estimated NOAEL. OHA applied a total UF of 10 for database limitations, since this is the only study of its kind for saxitoxin. For humans, no UF for interspecies variability was needed since the data were from human illnesses. OHA also did not apply an UF for individual variability since the EFSA study covered the general population, which included sensitive individuals. Using the acute RfD (0.5 µg/kg-day) as the POD and applying a total UF of 10 as shown in Equation (1), OHA calculated a human TDI of 0.05 µg/kg-day.

For dogs, OHA applied an additional UF of 10 for interspecies variability since dogs may be more sensitive to saxitoxins than humans. Therefore, using Equation 1 and a total UF of 100 for dogs, OHA calculated a dog-specific TDI of 0.005 µg/kg-day.

### 2.3. Review of Guideline Values Developed by Other Jurisdictions

For each of the four cyanotoxins, OHA reviewed guideline values developed by other states and nations and the methods used to derive those guidelines. According to the EPA, twenty states, including Oregon, have some kind of program or protocol to respond to HABs [18]. Of these twenty, fifteen include recreational guideline values for cyanotoxins as part of their response protocol [18]. Only three states, including Oregon, have guideline values for cyanotoxins in drinking water [18] and only California and Oregon have guideline values for dogs. The detailed results of this review are presented for each cyanotoxin in the Discussion section. 

## 3. Results

### 3.1. Guideline Values

Using the exposure assumptions shown in Table 1 and Equations (2) and (3), OHA calculated provisional drinking water and recreational water guideline values for humans and recreational guideline values for dogs for anatoxin-a, cylindrospermopsin, microcystins (microcystin-LR), and saxitoxins (SXT-eq) as summarized in Table 2. 

**Table 2:** Summary of Oregon’s tolerable daily intakes and guideline values for four cyanotoxins for use in acute or short-term exposures.

Guideline value	Anatoxin-a	Cylindrospermopsin	Microcystin	Saxitoxin
Human TDI (µg/kg-day)	0.1	0.03	0.05	0.05
Dog TDI (µg/kg-day)	None—used human TDI	None—used human TDI	None—used human TDI	0.005
Drinking Water (µg/L)	3.0	1.0	1.0	1.0
Recreational Water (µg/L)	20.0	6.0	10.0	10.0
Dog-specific (µg/L)	0.4	0.1	0.2	0.02

For drinking water, recreational water, and dog-specific guideline values, OHA rounded calculated results either to the nearest whole number or, if less than or equal to 0.5, to the nearest non-zero, post-decimal digit. Calculated drinking water values for microcystin and saxitoxin were both 1.5 µg/L, and OHA rounded down to 1.0 µg/L for public health protection. OHA used rounding to make the guideline values easier for partners to use and to avoid implying a degree of precision that does not exist.

### 3.2. Toxin-Based Monitoring Results in Oregon

Health-based guideline values for cyanotoxins allow OHA’s recreational HABs advisory program to make use of toxin-based monitoring (TBM) data. The alternatives to TBM in Oregon and in other states involve comparing cyanobacterial cell counts against WHO threshold values or even presence/absence of visible surface scum. Many species of cyanobacteria do not produce toxins, and even those strains that can produce toxins often do not. To be protective of health in the absence of toxin data, public health agencies must assume that potentially toxigenic species over a threshold cell count must be producing toxins at dangerous concentrations. This means that health advisories based on cell count or visible scum alone may often be unnecessary. 

Starting in 2012, OHA began encouraging partner agencies to adopt TBM approaches to water bodies that they manage throughout the state. OHA continued to issue health advisories based on cell counts or toxin data, depending on which method the partner agency selected. OHA asked partner agencies using a TBM approach to identify the dominant species in the bloom. This allowed partner agencies to focus testing on those toxins relevant to the species present. Partners sampled every other week throughout the life of the visible bloom. If toxins never exceeded recreational guideline values, OHA did not issue advisories, even if cell counts were over the threshold. If a toxin result exceeded a guideline value, OHA issued an advisory based on the toxin result and partner agencies stopped testing until all visible signs of the bloom were gone. At that point, partners collected confirmatory samples to verify that toxin levels and cell counts were below guideline values, and OHA lifted the advisories. 

**Figure 1:**
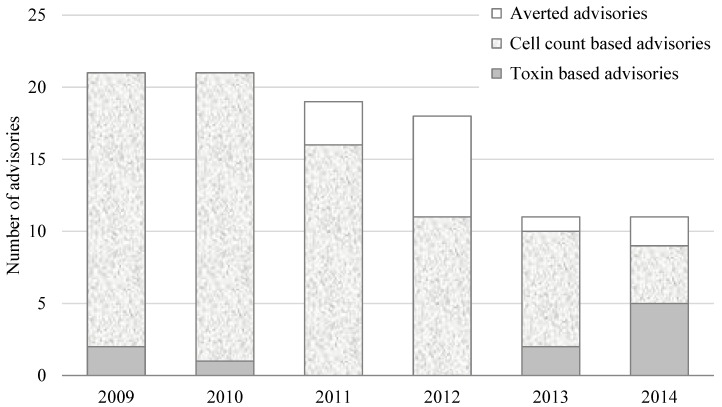
Number of advisories based on cell counts and toxin levels and number of potential advisories with cell counts above threshold that were averted by toxin tests with results below recreational guideline values. Recreational advisory thresholds are 100,000 cells/mL for all toxigenic genera except *Microcystis* and *Planktothrix*, for which the combined threshold is 40,000 cells/mL. Recreational guideline values for cyanotoxins are shown in Table 2.

Prior to the 2012 bloom season, most partner agencies only tested for cell counts and OHA issued advisories when cell counts were above the advisory threshold (100,000 cells/mL for any combination of potentially toxigenic genera or 40,000 cells/mL for any combination of *Microcystis* or *Planktothrix*) [1]. Figure 1 shows Oregon’s recreational advisories from 2009 to 2014 by year and basis for advisory (*i.e.*, cell counts *vs.* toxin results). Figure 1 also shows blooms that would have resulted in advisories based on cell count data had TBM not been employed showing toxins below guideline values. 

In total, OHA issued 88 recreational HABs advisories from 2009 to 2014. Of those, 78 were based on cell counts and 10 on toxin results. A total of 13 advisories were averted in the same time period where cell counts would have resulted in advisories had toxin results not been below recreational guideline values. Averted advisories shown in Figure 1 include only those HABs where cell counts were above advisory thresholds. 

In many cases, monitoring partner agencies noted visible HABs, identified the dominant genera present and went directly to toxin analysis without counting cells. In these cases, toxin levels were frequently below recreational guideline values. By these criteria, additional advisories likely averted by TBM were 12 in 2012, 14 in 2013, and 13 in 2014, though OHA cannot verify that these would have been advisories without cell counts. TBM did not appear to significantly change the mean time under advisory (8.0 weeks for cell count based advisories and 7.8 weeks for toxin based advisories).

**Figure 2:**
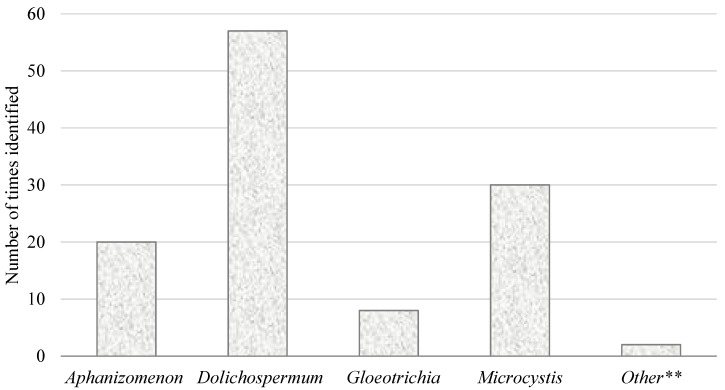
Number of times toxigenic genera of cyanobacteria were identified above cell count thresholds during advisories in monitored waterbodies, 2009–2014. Recreational advisory thresholds are 100,000 cells/mL for all toxigenic genera except *Microcystis* and *Planktothrix*, for which the combined threshold is 40,000 cells/mL. ** Other: *Phormidium* = 1, *Oscillatoria* = 1.

TBM also provided a clearer understanding of which cyanobacteria genera and toxins posed the greatest risk in Oregon. Figure 2 shows the number of times, from 2009 to 2014, toxigenic genera of cyanobacteria were counted above recreational advisory thresholds. *Dolichospermum* (formerly *Anabaena*) was the genus most frequently found over threshold, followed by *Microcystis* and more distantly by *Aphanizomenon* and *Gloeotrichia*. Cell counts were found above threshold 117 times during 88 advisories from 2009 to 2014. During 28 of those 88 advisories there were multiple genera counted over the threshold. In 18 out those 28 advisories, two genera were over the threshold. In six advisories, three genera were over the threshold, and in one advisory, four genera were over the threshold.

**Figure 3:**
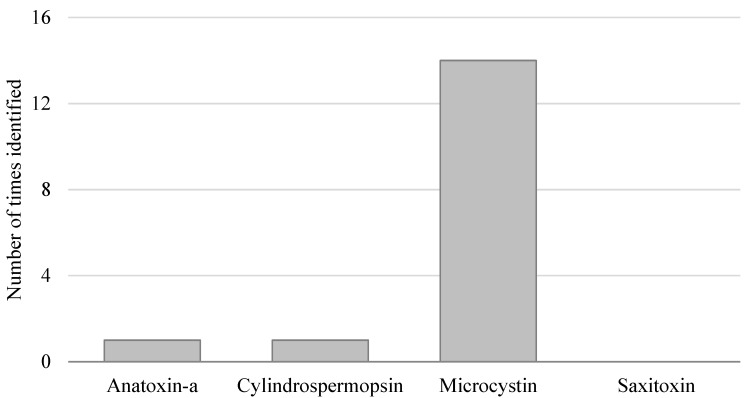
Number of times cyanotoxins were identified over human recreational guidelines during advisories in monitored waterbodies, 2009–2014. Guideline values are shown in Table 2.

Figure 3 shows the number of times cyanotoxins were detected over the recreational guideline values from 2009 to 2014. Microcystins are the cyanotoxins that most frequently exceed recreational guideline values in Oregon. Microcystins are also the most frequently detected cyanotoxins in Oregon, although all four cyanotoxins have been detected—saxitoxins being the rarest. 

In one case, two cyanotoxins (cylindrospermopsin and microcystin) were both measured above their recreational guideline values. In five cases, advisories were issued based on cell count data with toxin data coming in above the recreational guideline value after the advisory had already been established. 

In those five cases, multiple genera were counted above their advisory threshold and follow-up cyanotoxin levels were very high. For example, in one case total microcystins were measured at 705 µg/L at the same time *Microcystis*, *Dolichospermum*, and *Aphanizomenon* species were all present over their advisory cell count thresholds. 

In another case, total microcystins were measured at 310 µg/L when *Dolichospermum*, *Microcystis*, *Aphanizomenon*, and *Oscillatoria* were all counted above their advisory cell count thresholds. These data suggest that the presence of multiple toxigenic genera over threshold increases the likelihood of cyanotoxins over recreational guideline values and potentially far exceeding those values. 

## 4. Discussion

### 4.1. Intended Application of Guideline Values

Oregon’s cyanotoxin guideline values (summarized in Table 2) are intended to be applied to acute or short-term exposures. All of the studies from which Oregon derived TDIs pertain to exposures that are subchronic, short-term or acute. The lack of chronic toxicity data prevented OHA from developing chronic guideline values, and OHA continues to survey the literature and the work of regulatory agencies for useable chronic values. Low-dose, chronic exposure to cyanotoxins can have significant adverse health effects [1,27,28], and the absence of chronic guideline values should not imply that chronic exposure to cyanotoxins does not occur in Oregon. Many HABs in Oregon last for several weeks or months, posing the risk of prolonged or multiple intermittent exposures to a single HAB. Oregonians may frequent multiple bodies of water creating the potential for individuals to encounter multiple HABs over the course of a season and over many years. OHA does not have the resources or depth of Oregon-specific cyanotoxin concentrations over the course of a bloom or season to support a reliable or meaningful assessment of the risk to Oregonians from potential chronic exposure to cyanotoxins. Future research in this area would be extremely valuable if resources were made available.

OHA uses guideline values developed for specific structural variants of families of cyanotoxins to apply to the entire family. For example, OHA uses the guideline values developed based on microcystin-LR studies for all microcystins, and the guideline values developed for SXT-eq to apply to total saxitoxins. Many of OHA’s partner agencies test for microcystin and saxitoxin using enzyme linked immunosorbent assays (ELISA), which cross-react with multiple variants within the families. Therefore, it is important for OHA to be able to use guideline values in the context of total microcystins or total saxitoxins as measured by ELISA. A limited number of studies comparing the *in vivo* toxicity of three of the more commonly detected variants (microcystins-LR, RR, YR) indicate that microcystin-LR is the most toxic of those three [28,29]. *In vitro* studies indicate that microcystins-LF and LW might be more toxic *in vitro*, perhaps because these variants are more lipophilic and enter cells more readily in an *in vitro* setting [30]. This hypothesis has not been tested *in vivo*. Overall, these studies suggest that application of the microcystin-LR-based guideline values to total microcystins is health protective. EFSA’s TEF analysis [26] suggests that OHA’s guideline values for saxitoxins (SXT-eq) provide a health protective approach.

Dog-specific guideline values are not used as a basis for issuing recreational HAB advisories. These values are used to communicate the significance of cyanotoxin exposures with veterinarians and pet owners to help reduce dog illnesses and death. OHA has outreach and educational materials targeted at pet owners that are available to the public and posted at monitored water bodies in Oregon. 

Drinking water guideline values are not used as a basis for issuing recreational HAB advisories. These values are used by drinking water staff to determine if a cyanotoxin problem exists in raw or finished water samples taken from public drinking water facilities where a bloom has been identified in source water. These samples are taken weekly throughout the life of the bloom. Although raw water samples have tested as high as 5.24 µg/L for microcystin-LW, Oregon has not had finished drinking water samples with cyanotoxin levels above drinking water guideline values. The highest detection of any cyanotoxin in finished drinking water was 0.3 µg/L anatoxin-a. 

### 4.2. Uncertainties and Limitations Surrounding Exposure Assumptions for Calculation of Guideline Values

When calculating cyanotoxin guideline values for Oregon, OHA selected default assumptions for inputs such as body weight and water consumption from standard government agency sources. While Oregon’s guideline values are designed to be representative of the general population, it would of course be more accurate to use population-specific measurements for such inputs. Unfortunately, OHA did not have resources to develop Oregon-specific inputs for derivation of guideline values. 

OHA exercised professional judgment in selecting the ages of four to six years as the youngest likely to swim for hours at a time. Of course, other ages could have been selected and different guidelines calculated for those alternate selections, but OHA considers the assumptions selected to be realistic and protective of public health. 

Studies have shown that cyanotoxins, microcystins in particular, can be found in fish tissue [1,31], though at lower levels in the fillet than in lipophilic tissues. Cyanotoxins may also be found in crops irrigated with affected water [32]. Contaminated fish or crops could serve as an additional source of cyanotoxin exposure to people who consume them. This possibility could mean that an RSC other than 1 should be selected when calculating drinking water guideline values. However, exposure to cyanotoxins via food sources would be expected to constitute a more chronic exposure, and OHA’s guideline values are focused on short-term, acute exposures. 

### 4.3. Limitations and Uncertainties in Selection of Critical Toxicity Studies for TDI Derivation

#### 4.3.1. Anatoxin-a

Very few applicable studies have been conducted with the intent to identify dose-response relationships to anatoxin-a administered orally. Therefore, OHA applied an UF for database limitations in the TDI derivation to account for possible future studies that may reveal that anatoxin-a has different toxicity than has been suggested in currently available literature.

OHA identified only two primary studies that employed oral administration of anatoxin-a: the Fawell, *et al.*, study, selected as the critical study [13,14] and an older study conducted by Astrachan, *et al.* [7,8]. Independent reviews [10,11] of the Astrachan study have derived a TDI of 0.51 µg/kg-day, a value within a factor of 5 to the TDI selected (0.1 µg/kg-day). CALOEHHA has proposed a subchronic oral reference dose (RfD) of 2.5 µg/kg-day [6], consistent with EPA’s proposed RfD [20] that did not consider the death in the 500 µg/kg-day dose group to be treatment related in the critical study [13,14]. Other toxicity studies [16] have been conducted using non-oral routes of exposure (mainly intraperitoneal injection). Because human exposure to anatoxin-a in Oregon is expected to be primarily through ingestion, either in drinking water or accidental ingestion of surface water while recreating, OHA considered only those studies using oral routes of exposure. 

#### 4.3.2. Cylindrospermopsin

The EPA’s 2006 toxicological review of cylindrospermopsin included a comprehensive summary of toxicological studies collected up to that time [23]. Since then a few relevant studies and reviews have been published [27,33]. However, OHA relied heavily on the EPA’s 2006 review, because it is robust and comprehensive and because newer studies have not identified lower subchronic NOAELs than those described in EPA’s 2006 review. 

One study [27] by Sukenik, *et al*., evaluated chronic (42-weeks) exposure in mice via drinking water and identified a LOAEL of 20 µg/kg-day based on increased hematocrit and deformed erythrocytes. This study did not include as many endpoints as the Humpage *et al.*, study [24] and exposed the same experimental groups of animals to gradually increasing doses of cylindrospermopsin, rather than treating separate groups of animals with consistent doses throughout the experiment. This dosing regimen poses difficulties in interpretation for either chronic or short-term exposure effects for the purposes of TDI development. Lower doses early in the experiment could have created tolerance in the experimental group, which could have made them resistant to higher doses later. For these reasons, OHA could not justify the use of this study in support of either a chronic TDI or chronic guideline values. 

#### 4.3.3. Microcystins

Two different critical studies have been used by different entities to develop TDIs or RfDs for microcystin-LR. The WHO used a study conducted by Fawell, *et al.* [34]. In this study, mice were dosed by oral gavage for 13 weeks. The study identified a NOAEL of 40 µg/kg-day. WHO divided this NOAEL by a total UF of 1000 to develop a TDI of 0.04 µg/kg-day. The total UF is 10 to account for interspecies variability, 10 for individual variability and 10 for limitations in the database, particularly surrounding cancer and chronic disease and reproductive health endpoints. 

The second critical study was conducted and published by R. Heinze [25], which OHA used as the basis for its TDI. CALOEHHA also chose the Heinze study because “…it evaluated more endpoints, utilized a better experimental design, showed greater target organ specificity (intrahepatic hemorrhage) in the histopathological analysis, and showed a clear dose-response trend” when compared with other toxicity studies [6]. 

CALOEHHA applied benchmark dose (BMD) techniques to determine a BMD of 6.4 µg/kg-day [6]. They then applied an UF of 1000 to establish an acute RfD of 0.006 µg/kg-day. The total UF consisted of 10 for interspecies variability, 10 for individual variability, and 10 for limitations in the database, particularly surrounding cancer and reproductive health endpoints. 

The rats of the Heinze study were also more sensitive to microcystin-LR than the mice of the Fawell study, and general toxicological practice is to use the most sensitive endpoint and species as the basis for selecting a critical study.

OHA agreed with CALOEHHA’s selection of the critical study and the selection of the 50 µg/kg-day LOAEL as the basis for development of an acute TDI. However, guidance for using BMD techniques [35] recommends against using it in cases where there are fewer than three dose groups (not counting controls) and the Heinze study only had two dose groups. Instead of BMD, OHA applied an UF of 10 to achieve an estimated NOAEL of 5 µg/kg-day as is consistent with EPA guidance [36]. It is worth noting that the difference between using BMD and the UF to adjust down from the LOAEL is only 1.4 µg/kg-day and the UF is slightly more protective.

The TDI developed by WHO (0.04 µg/kg-day), based on the Fawell study [34] is very similar to the provisional acute value (0.05 µg/kg-day) proposed here. CALOEHHA’s selection [6] of the Heinze study [25] also supports OHA’s decision to use the same study. A chronic (18 month) mouse toxicity study of microcystin-LR in drinking water identified a NOAEL of 3 µg/kg-day [37]. This number is very similar to the estimated 5 µg/kg-day NOAEL OHA used to develop the state’s provisional TDI based on the Heinze study.

#### 4.3.4. Saxitoxins

OHA was unable to find any additional relevant studies for use in TDI development for saxitoxins. Therefore, the application of the UF for database limitations is especially important in this case.

### 4.4. Comparison with Guideline Values Developed by Other Nations and States

#### 4.4.1. Anatoxin-a

The New Zealand Ministry of Health has a drinking water guideline (6 µg/L) [21] for anatoxin-a. Duy, *et al.* [11] proposed a drinking water guideline value of 2.72 µg/L for infants, 4.08 µg/L for children and 12.24 µg/L for adults. Oregon’s value falls near the most protective end of this range. Codd, *et al.* [10], in an independent review of the literature, proposed supporting the 12.24 µg/L value put forward by Duy, *et al.* for adults. Ohio has a drinking water guideline of 20 µg/L [22]. 

CALOEHHA has also proposed a recreational water guideline value for swimmers derived using a higher TDI (2.5 µg/kg-day). However, the result (90 µg/L) [6] is similar, within a factor of 4.5 to the recreational water guideline value developed for Oregon (20 µg/L). Washington State Department of Health adopted 1 µg/L as their guidance value for recreational water [19]. Ohio has a recreational guideline value of 80 µg/L with a no contact advisory at 300 µg/L [18]. Vermont has a guideline value of 10 µg/L for anatoxin-a [18].

#### 4.4.2. Cylindrospermopsin

New Zealand’s Ministry of Health has drinking water guidelines for cylindrospermopsin equal to OHA’s provisional guideline of 1 µg/L [21]. Ohio also has a drinking water guideline value of 1 µg/L [22]. 

OHA’s provisional recreational water guideline value is similar to those proposed by other governmental bodies. CALOEHHA proposed a guideline value of 4 µg/L [6]. The Department of Health for Washington State has proposed a recreational guideline value of 4.5 µg/L [19]. Ohio has a recreational guideline value of 5 µg/L with a no-contact advisory level of 20 µg/L [18]. These values are similar to the provisional recreational water guideline value developed for Oregon (6 µg/L). 

#### 4.4.3. Microcystins

OHA’s provisional drinking water guideline for microcystin-LR (1 µg/L) is identical to the drinking water guideline established by the WHO [1] and the actual calculated value of 1.5 µg/L is identical to the value finalized by Health Canada [3]. Ohio also has a drinking water guideline of 1 µg/L [22] and Minnesota has a drinking water guideline of 0.04 µg/L [18]. 

OHA’s recreational water guideline value (10 µg/L) is the same as the upper limit of “mild and/or low probability of adverse health effects” suggested by the WHO [1]. Illinois also has a recreational guideline value of 10 µg/L [18], slightly higher than the Washington State guideline of 6 µg/L [18], which is also shared by Vermont and Virginia [18]. OHA’s value is 12.5 times greater than California’s recreational value of 0.8 µg/L [6]. Indiana and Kansas have tiered systems that use 4 µg/L as a threshold for recreational activities and 20 µg/L as a threshold for any water contact [18]. Ohio also has a tiered system using 6 and 20 µg/L as the guideline values [18]. Iowa, Nebraska, Oklahoma, and Texas use a 20 µg/L value [18]. Massachusetts and Rhode Island use 14 µg/L as a recreational guideline value for microcystin [18].

#### 4.4.4. Saxitoxins

New Zealand has a recommended drinking water guideline value for STX of 3 µg/L [21]. Ohio has a drinking water guideline value of 0.2 µg/L [22]. The Ohio drinking water guideline is also based on the EFSA acute RfD, to which they applied an additional UF of 10 for individual variability. OHA did not consider this UF necessary because the study included sensitive individuals in the general population. No other states have drinking water guideline values for saxitoxin. 

Washington also used EFSA’s acute RfD as the basis for their recreational water guidance value of 75 µg STX-eq/L [19]. Oregon’s value is different from Washington’s because OHA chose to use 20 kg as the assumed body weight of a child while Washington used 15 kg. In addition, Washington did not apply the UF for database limitations to the EFSA acute RfD. Ohio uses a tiered system of 0.8 µg/L for recreational contact and 3 µg/L to avoid all contact [18]. 

### 4.5. Additional Cyanotoxins

The four cyanotoxins for which OHA has developed guideline values are a small fraction of the cyanotoxins and other metabolic products of cyanobacteria. When a person swallows water while swimming in HAB-affected surface water, they likely ingest entire cyanobacterial organisms including everything they produce and contain. When this happens, additional components of the cyanobacterial cells may increase toxicity as compared to exposure to the purified toxin alone [23,33]. How additional cyanobacterial components may increase the toxicity of known cyanotoxins has not been adequately characterized in the literature. Therefore, OHA was not able to quantitatively adjust guideline values to account for these potential increases in toxicity. 

OHA focused efforts on the cyanotoxins most frequently detected in Oregon. However, there is always the risk that additional toxins could be present for which no water body managers in Oregon are currently monitoring. For example, nodularin is another hepatotoxic cyanotoxin produced by cyanobacteria of the *Nodularia* genus [1]. Nodularin has never been monitored for in Oregon, so its frequency cannot currently be determined. However, *Nodularia* organisms have never been reported above their cell-count threshold in Oregon, so this would indicate that nodularin is not currently posing significant risk to public health in the state. 

All cyanobacteria produce lipopolysaccharides (LPS) as a structural component of their cell wall [1]. For allergic or sensitive individuals, dermal exposure to LPS can cause skin rashes. However, such rashes only affect a small segment of the public, are self-limiting (*i.e*., do not require medical attention) and lack a dose-response relationship [38]. Without a dose-response relationship it is impossible to identify a threshold at which an LPS-based advisory should be issued. For this reason OHA does not base health advisories on the risk of LPS exposure alone. 

Another common metabolic product of cyanobacteria is α-amino-β-methylaminoproprionic acid (BMAA). Most cyanobacteria produce this compound, which has been implicated in increased risk of neurodegenerative diseases such as amyotrophic lateral sclerosis and Parkinson’s disease in human epidemiological studies [39,40]. Increasing evidence indicates that BMAA can cause these illnesses following chronic exposure, by mimicking the amino acid L-serine, causing misfolded proteins in brain cells [41]. Clear dose-response relationships have not yet been established. In the absence of definitive data on dose-response relationships, OHA is unable to create guideline values for BMAA, and as a result may be underestimating the public health risk from cyanobacterial blooms in Oregon. 

For all of the reasons above, monitoring programs that exclusively focus on cyanotoxins could underestimate risk to public health. However, the overall weight of evidence suggests that most of the risk for illness and disease associated with cyanobacteria can be averted by avoiding contact with the dominant cyanotoxins that have been identified. 

### 4.6. Benefits of Toxin Based Monitoring

Toxin based monitoring benefits Oregonians in multiple ways. Expenditure of public health resources can be focused on those blooms with greatest risk to the population. Toxin data allow OHA to communicate with the public about actual risks, as opposed to the potential risk represented by cell count data alone. Toxin data give greater credibility to health advisories when they are issued and decrease the likelihood that an advisory would be issued unnecessarily. 

Our results indicate that TBM decreases the likelihood that an advisory will be issued. This allows Oregonians to enjoy more outdoor recreation, increasing physical activity and strengthening social networks without the frustration of cancelling plans or losing deposits related to unexpected HABs advisories. Decreased advisories also reduce the risk of “advisory fatigue” wherein people stop heeding advisories because they are so frequent that they do not notice them anymore. 

Although OHA does not close lakes when recreational advisories are in effect, reports from partner agencies that manage these waterbodies indicate that many fewer Oregonians visit during advisories. This anecdotal evidence is supported by national studies from various waterbody types around the country that suggest significant economic burdens on tourism industries when HABs-related health advisories are in effect [42,43,44,45]. If accurate, this decrease in visitors is an economic hardship on businesses that depend on the water tourism industry. Therefore, using TBM approaches may also reduce the economic burden of HABs by focusing advisories on those water bodies that need them and avoiding them where they are not needed. 

## 5. Conclusions

Cyanotoxins have the potential to harm public health and they are present in Oregon’s fresh waters. Health-based guideline values for cyanotoxins are necessary to evaluate risks posed from cyanobacterial blooms. To address this need, Oregon established guideline values for anatoxin-a, cylindrospermopsin, microcystins, and saxitoxins. Guideline values include acute tolerable daily intakes (TDI), human drinking and recreational water and dog-specific guideline values. 

Application of guideline values allowed for meaningful TBM of HABs-affected waterbodies in Oregon. TBM reduced the number of advisories, which benefited public health and the local tourism-based economy. 

## Abbreviations

ATSDR: Agency for Toxic Substances and Disease Registry
BMAA: α-amino-β-methylaminoproprionic acid
BMD: Benchmark Dose
BW: Body weight
BWIR: Body weight normalized intake rate
C: C-toxins
CALOEHHA: California EPA’s Office of Environmental Health Hazard Assessment
dcSTXs: 11-hydroxy-STX and decarbamoylsaxitoxins
EFSA: European Food Safety Authority
ELISA: enzyme linked immunosorbent assay
EPA: U.S. Environmental Protection Agency
GTX: Gonyautoxins
HAB: Harmful algae bloom
IR: Intake rate
LOAEL: Lowest observable adverse effect level
LPS: Lipopolysaccharide
neoSTX: Neosaxitoxin
NOAEL: No observable adverse effect level
OHA: Oregon Health Authority
POD: Point of departure
RfD: Oral reference dose
RSC: Relative source contribution
STX: Saxitoxin
STX-eq: Saxitoxin equivalent
TBM: Toxin based monitoring
TEF: Toxic equivalency factor
TDI: Tolerable daily intake
UF: Uncertainty factor
WHO: World Health Organization
